# S1P Signaling Pathways in Pathogenesis of Type 2 Diabetes

**DOI:** 10.1155/2021/1341750

**Published:** 2021-01-19

**Authors:** Qiong He, Jiaqi Bo, Ruihua Shen, Yan Li, Yi Zhang, Jiaxin Zhang, Jing Yang, Yunfeng Liu

**Affiliations:** ^1^Department of Endocrinology, First Hospital of Shanxi Medical University, Taiyuan 030001, Shanxi Province, China; ^2^Department of Second Medical College, Shanxi Medical University, Taiyuan 030001, Shanxi Province, China; ^3^Department of Pharmacology, Shanxi Medical University, Taiyuan 030001, Shanxi Province, China; ^4^Key Laboratory of Cellular Physiology, Ministry of Education, Shanxi Medical University, Taiyuan 030001, Shanxi Province, China

## Abstract

The pathogenesis of type 2 diabetes mellitus (T2DM) is very complicated. The currently well-accepted etiology is the “Ominous Octet” theory proposed by Professor Defronzo. Since presently used drugs for T2DM have limitations and harmful side effects, studies regarding alternative treatments are being conducted. Analyzing the pharmacological mechanism of biomolecules in view of pathogenesis is an effective way to assess new drugs. Sphingosine 1 phosphate (S1P), an endogenous lipid substance in the human body, has attracted increasing attention in the T2DM research field. This article reviews recent study updates of S1P, summarizing its effects on T2DM with respect to pathogenesis, promoting *β* cell proliferation and inhibiting apoptosis, reducing insulin resistance, protecting the liver and pancreas from lipotoxic damage, improving intestinal incretin effects, lowering basal glucagon levels, etc. With increasing research, S1P may help treat and prevent T2DM in the future.

## 1. Introduction

Approximately 9% of adults have diabetes worldwide, and approximately 90% of patients with diabetes have type 2 diabetes mellitus (T2DM) [1]. T2DM is a chronic disease that seriously endangers human health; thus, it is imperative to improve our understanding of T2DM.

**Figure 1:**
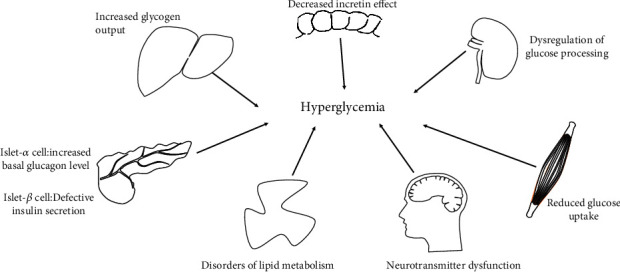
Ominous Octet theory in diabetes.

The pathogenesis of T2DM is complex. One of the most acceptable pathogeneses of T2DM is the “Ominous Octet” theory proposed by Professor Defronzo [2] (Figure 1). “Ominous Octet” is a supplement to the previous “Triumvirate” diabetes pathogenesis theory. The updated theory states that the mechanism of T2DM can be provided in the following eight aspects: (1) defects in islet *β* cell secretion, including endoplasmic reticulum stress, mitochondrial dysfunction, and inflammation, together lead to islet *β* cell failure [3]; (2) decreased glucose uptake in muscle tissue: a high-sugar and high-fat environment destroys the insulin signaling pathway in muscle tissue; thus, glucose uptake is reduced [4, 5]; (3) increased liver glycogen output: hepatic glucose production is enhanced; thus, glucose levels are increased [6]; (4) disturbance of lipid metabolism: excessive free fatty acids mediate insulin resistance in the liver and pancreas [7]; (5) impaired incretin effect: GLP-1 deficiency leads to decreased insulin secretion and increased glucagon secretion [8]; (6) basal glucagon levels are elevated, causing the levels of fasting blood glucose to increase [9]; (7) dysregulation of glucose in the kidneys: the dysfunction of kidney-sodium-dependent glucose transporter 2 and other molecules leads to the disordered reabsorption of glucose [10]; (8) neurotransmitter dysfunction: the inhibitory effect of the appetite is weakened and excessive energy intake triggers T2DM [11]. For patients with T2DM, these eight pathogenic factors can exist alone or intertwined, eventually leading to the development of T2DM.

Sphingosine 1 phosphate (S1P) is a protective lipid molecule for T2DM. In the central nervous system, S1P suppresses appetite by inhibiting related signal transmission. In peripheral organs, S1P has an effect on resisting inflammation and oxidative stress, delaying the progress of insulin resistance, enhancing GLP-1 secretion and effects, and protecting the kidneys. Based on various studies of S1P in recent years, S1P and its related molecular signaling pathways play a crucial role in the multiple pathogenesis of T2DM. S1P is an emerging research hotspot for the prevention and treatment of T2DM. This review summarizes the physiological and pharmacological effects of S1P in the pathogenesis of T2DM to provide new research ideas for future studies.

## 2. Overview of S1P

S1P is a lysophospholipid with various biological activities. The synthesis of S1P begins with sphingomyelin and glycosphingolipids, both of which are localized in the lysosome and plasma membrane microregions. Sphingomyelin and glycosphingolipid degrade to form the intermediate product ceramide, ceramide then converts to sphingosine, and sphingosine is finally phosphorylated by sphingosine kinase 1 and 2 to form S1P [12, 13]. When S1P is synthesized, it is decomposed into hexadecenal (Hex) and phosphoethanolamine (PE) by S1P lyase and other specific S1P phosphohydrolases. Hex and PE then enter the biosynthetic pathway of triglyceride [14]. S1P is also dephosphorylated and enters the ceramide synthesis pathway [15].

**Figure 2:**
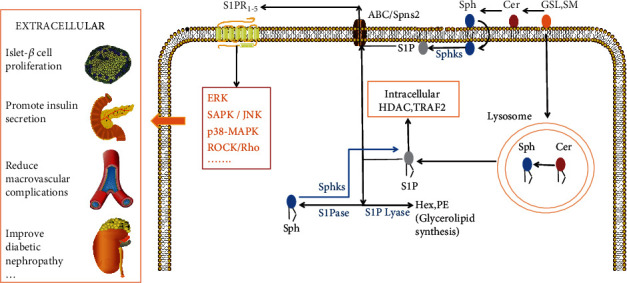
S1P metabolic pathway.

Ceramide is converted to sphingosine, and sphingosine is phosphorylated to form S1P; conversely, S1P is dephosphorylated and catalyzed by ceramide synthase to form ceramide. The metabolic pathway between ceramide and S1P is called “sphingolipid rheostat” [12]. The synthesis and decomposition of S1P in cells are strictly regulated in time and space [16]. The main targets of intracellular S1P are histone deacetylase, TNF receptor-associated factor 2, and Spns2 transporters [17, 18]. In addition, S1P can be transported to the outside of cells through the ABC/Spns2 transporter to act on the S1P receptor (S1PR). After combination with S1PR1-5, S1P mainly activates ERK, SAPK/JNK, p38-MAPK, and ROCK/Rho signaling pathways to regulate the proliferation of islet *β* cells, promote insulin secretion, reduce the complications of large vessels, and relieve diabetic nephropathy (see Figure 2 for details).

Extracellular S1P binds to S1PR_1~5_ on the cell surface and activates downstream signaling pathways. S1PRs are part of the G protein-coupled receptor family.

S1PR_1_ can activate intracellular regulatory protein kinase (ERK) through Gi [19] and inhibit adenylate cyclase [20]. S1PR_2_ and S1PR_3_ are mainly coupled with Gq to activate phospholipase C (PLC), which induces Ca^2+^ mobilization [21, 22] and vitalizes ERK, c-Jun amino terminal kinase (JNK), p38-mitogen-activated protein kinase (p38-MAPK) [23], and Rho-associated protein kinase/Rho protein (ROCK/Rho) [24]. Activation of these pathways regulates cell growth, differentiation, and proliferation as well as inhibits inflammation and apoptosis. The mechanisms of S1PR_4_ and S1PR_5_ have not yet been fully clarified (see Table 1 for details).

## 3. The Role of S1P in the Pathogenesis of T2DM from the View of the Ominous Octet

### 3.1. S1P Protects *β* Cell Function

#### 3.1.1. S1P Promotes *β* Cell Proliferation and Reduces Apoptosis

The decrease in the number of islet *β* cells is a hallmark of T2DM. Increasing the concentration of S1P or upregulating the expression of S1PR has a hypoglycemic effect by increasing the number of *β* cells and insulin levels.

FTY720, a S1PR agonist, can effectively promote pancreatic *β* cell proliferation as well as inhibit its apoptosis in T2DM mice. The S1P signaling pathway promotes proliferation by activating intracellular phosphatidylinositol 3 kinase (PI3K), regulating cyclin D3 and p57KIP2, and mediating *β* cell regeneration [25].

In addition, studies regarding islet *β* cell apoptosis have confirmed that the diabetic mice treated with S1P significantly reduced glucose tolerance level, insulin resistance, and the number of *β* cell apoptotic cells compared with the untreated group. These results indicated that S1P has a protective effect on *β* cells. In addition, S1PR_1_ and S1PR_2_ expression in the S1P treatment group significantly increased, indicating that S1P plays a role in antagonizing *β* islet cell failure by upregulating S1PRs [26].

#### 3.1.2. S1P Delays *β* Cell Failure by Improving Mitochondrial Function

Chronic hyperglycemia and hyperlipidemia are the main factors leading to *β* cell energy metabolism damage, which mainly injures the normal function of the mitochondrial membrane, cytochrome c release, and caspase-3 activation, leading to *β* cell failure [27–29].

The mitochondrial unfolded protein response (UPR) is a mechanism that maintains mitochondrial homeostasis in an adverse environment. UPR, activated by misfolded or unfolded protein accumulation, upregulates the translation of molecular chaperones and proteases, thereby maintaining protein homeostasis [30]. When UPR occurs, sphingosine kinase activates then catalyzes sphingosine to produce S1P. The newly formed S1P may be involved in the initiation of UPR in the cytoplasm or the assembly of proteins in mitochondria [31]. S1P amplifies the protective function of UPR on *β* cells under stress conditions, such as hyperglycemia and hyperlipidemia.

#### 3.1.3. S1P Antagonizes *β* Cell Apoptosis

Extracellular S1P can antagonize inflammatory cytokine-induced mitochondrial cytochrome c release and inhibit caspase-3 activity in rat pancreatic islet cells, suggesting that S1P can antagonize inflammatory factor-mediated *β* cell apoptosis [32].

### 3.2. S1P Improves the Negative Effects of Insulin Resistance in Muscle

Skeletal muscle is the major organ involved in glucose processing in the body. Impaired glucose transport in the skeletal muscle is an important cause of insulin resistance in T2DM. This defect can be attributed to abnormal insulin signal transduction in the skeletal muscle tissue. Insulin normally binds to insulin receptors on the plasma membrane of skeletal muscle cells, activates the Akt pathway, and improves glycogen synthesis and transport [33]. Ceramide can activate atypical protein kinase *ζ* (protein phosphatase *ζ* (PKC*ζ*)), phosphorylate Akt, reduce its affinity with phosphatidylinositol triphosphate, and reduce Akt recruitment to the plasma membrane [34, 35]. The upstream kinase 3-phosphoinositide-dependent protein kinase 1 (PDK1) and TORC2 must activate the Akt recruited to the plasma membrane to promote glucose utilization. Ceramide blocks the downward transmission of PDK1 and TORC2 signals and antagonizes the Akt signaling pathway [33]. Ceramide also activates protein phosphatase 2A, an enzyme associated with Akt dephosphorylation [36], which inhibits IRS-1 tyrosine phosphorylation [37] and attenuates downstream insulin signaling [38, 39]. High glucose levels directly stimulate ceramide catabolism and S1P formation [40], whereas S1P inhibits ceramide's function by blocking the Akt pathway.

#### 3.2.1. Ceramide Induces Insulin Resistance through Multiple Pathways

Ceramide blocks the Akt pathway, after which the effects of the Akt pathway on promoting glycogen synthesis and glucose transport are also impaired [36]. Ceramide inhibits the activation of insulin receptors. When treated with ceramide, insulin receptor tyrosine phosphorylation is reduced [39], leading to weakening of the insulin signal. Moreover, ceramide inhibits the translocation of glucose transporter protein 4 (GLUT4) to the cell membrane [41].

Furthermore, ceramide damages mitochondria, which leads to impaired glucose and fat utilization. The specific process is as follows: ceramide directly inhibits the electron transport of respiratory chain enzyme complex I and reduces the activity of enzyme complex III [37]. Ceramide increases the permeability of the mitochondrial membrane to form the ceramide channel and promotes ions and protein release [42]. Finally, the structure of mitochondria and the function of the respiratory chain are destroyed, resulting in impaired glucose and lipid utilization [43].

#### 3.2.2. S1P Improves the Negative Effects of Insulin Resistance in Muscle

The effect of S1P on ceramide is reflected in the sphingolipid rheostat, which is the metabolic pathway between ceramide and S1P. As mentioned in the overview [44], S1P can be degraded to ceramide, and ceramide can also generate S1P [12]. Ceramide induces insulin resistance. However, when S1P increases, that is, more ceramide converts to S1P, ceramide's effect of inducing insulin resistance is weakened. The balance between intracellular ceramide and S1P is essential for the development of cellular insulin resistance.

#### 3.2.3. S1P As a Key Factor Involved in the Function of Adiponectin Reversing Insulin Resistance

Adiponectin activates AMP-activated protein kinase (AMPK). The activation of AMPK promotes fatty acid oxidation and decomposition, reducing the level of ceramide [45, 46]. In addition, activated AMPK stimulates GLUT4 translocation and increases glucose transport [47, 48].

When adiponectin and insulin are combined, the tyrosine phosphorylation level of insulin receptors in muscle increases [49], which helps to improve insulin sensitivity. Adiponectin can also act as an autocrine and paracrine factor to inhibit the secretion of IL-6 and IL-8 inflammatory factors as well as chemoattractant protein. Moreover, adiponectin accelerates ceramide degradation and increases S1P concentration independent of the AMPK pathway.

S1P is a key factor in the antiapoptotic effect of adiponectin on pancreatic *β* cells. In in vitro experiments, adiponectin prevented ceramide accumulation and reduced ceramide-induced apoptosis; however, the addition of sphingosine kinase inhibitors counteracted the antiapoptotic effect. The results showed that adiponectin needs to rely on S1P to achieve its functions [40].

### 3.3. S1P Inhibits Hepatic Glucose Production

The liver is an important organ for regulating blood glucose concentration, and it is the main function that includes regulating glycogen synthesis and gluconeogenesis. When fasting plasma insulin levels increase by 2.5-3 times, the liver produces excessive glucose to compensate for insulin's lowering glucose function [2]. Furthermore, lipotoxicity and glycotoxicity can increase hepatic glucose output. For example, pyruvate carboxylase is the rate-limiting enzyme of gluconeogenesis but is overexpressed when lipids accumulate, resulting in high blood glucose [50, 51]. Lipotoxicity and glycotoxicity lead to enhanced gluconeogenesis and increased hepatic glucose output, which causes diabetes.

S1P inhibits hepatic glucose output by activating the Akt pathway. Experimental results have confirmed that sphingosine kinase enhances Akt phosphorylation in a dose-dependent manner [52]. Activation of the Akt signaling pathway plays the following roles: first, it increases the level of GLUT2 mRNA in hepatocytes and promotes the expression of GLUT2 in hepatocytes [53]; second, it promotes the expression of GLUT1 and GLUT3; third, phosphorylating phosphofructokinase 2 to promote glycolysis, thereby lowering blood glucose levels.

However, S1P improves UPR to rival cellular stress. The increase in S1P concentration, by knocking out the homologous Spgg1 gene, can improve the ER response and basal insulin secretion of various types of cultured cells [54]. In conclusion, S1P weakens the endoplasmic reticulum stress-mediated glucose output in the liver via the Akt pathway [55].

### 3.4. S1P Withstands the Damage Effects of Lipotoxicity

The prominent role of lipids in organisms is to store energy and function as the main component of cell membranes [56]. When the content of free fatty acids is higher than normal for a prolonged period of time, it damages the functions of important organs, such as the pancreas and liver. This effect is called lipotoxicity [3, 57].

Experiments at the pancreatic cell and tissue levels suggest that S1P maintains the normal activity of *β* cells and pancreatic tissue in a high-fat environment [32, 58]. Other animal experiments have further confirmed that under high-fat conditions, S1P can mobilize a large amount of insulin secretion and delay the occurrence of diabetes [59]. Feeding sphingosine kinase 1 gene-deficient and wild-type mice a high-fat diet results in sphingosine kinase 1 gene-deficient mice suffering from diabetes, while wild-type mice only show mildly impaired blood glucose regulation. Insulin levels in the circulation of wild-type mice approached three times that of sphingosine kinase 1 gene-deficient mice [59]. More experimental results have revealed that under high-fat conditions, the accumulation of a large number of reactive oxygen species is the main cause of cell damage, and S1P can fight cytotoxic substances produced during oxidative stress and play a protective role [60].

Lipid toxicity damages the normal structure and function of liver cells. Abnormal liver function aggravates the disorder of lipid metabolism, forming a vicious circle, which eventually induces diabetes. In Sphk1 gene-deficient liver cells, the proportion of liver cell death increases with the increase in lipotoxicity. In contrast, liver cells overexpressing the sphingosine kinase 1 gene are protected from lipotoxic damage, which may be related to S1P in reducing JNK phosphorylation and inhibiting the C/EBP homologous protein signaling pathway [61].

### 3.5. S1P Enhances the Incretin Effect

Incretin is a type of gut-derived hormone secreted by intestinal epithelial endocrine cells, mainly including glucose-dependent insulin-like polypeptide (GIP) and glucagon-like peptide 1 (GLP-1). Intestinal endocrine L and K cells secrete GLP-1 and GIP, respectively. After eating, food enters the intestine and stimulates cells to secrete secretin [62].

Oral glucose can stimulate more insulin secretion than intravenous glucose [63], and this effect is called the incretin effect. The incretin effect accounts for 60% of the total insulin release after a meal. When the incretin effect is weakened, insulin release decreases, causing postprandial blood sugar to rise and blood sugar fluctuations to increase, eventually leading to diabetes. GLP-1 and GIP are mainly included in incretin. For the effect on gut secretion of two types of incretin, GLP-1 plays a major role, while the effect of GIP is relatively weak. In patients with T2DM, the physiological function of GLP-1 is not affected. However, a significant reduction in secretion leads to T2DM [64].

S1P increases the secretion of GLP-1 by activating the PLC-PKC signaling pathway. Under normal circumstances, food intake activates the Gq protein-coupled receptor on intestinal L cells, enabling the G protein-PLC-PKC signaling pathway. S1P can activate the PLC pathway in this process, after which PLC catalyzes the hydrolysis of plasma membrane lipid phosphatidylinositol diphosphate to generate 1,4,5-triphosphate inositol (IP3) and diacylglycerol (DG). IP3 and DG, acting as second messengers, increase the Ca^2+^ concentration and activate PKC, respectively. Finally, the secretion of GLP-1 increases [65].

Activating the S1P signaling pathway not only promotes the secretion of GLP-1 but also produces the same biological effects as GLP-1. In pancreatic islet *β* cells, GLP-1 binds to its receptor to activate adenylate cyclase (AC), increase intracellular cAMP content, and initiate protein kinase A, triggering Ca^2+^ release and releasing insulin [66, 67]. GLP-1 can also improve the survival of islet *β* cells by activating the PI3K and Akt signaling pathways [68, 69]. S1P can directly activate pathways, such as AC and Akt, and exhibit physiological functions similar to those of GLP-1 [58].

### 3.6. S1P Inhibits Glucagon

The blood glucose balance is mainly regulated by glucagon and insulin. The high glucagon level causes an increase in blood glucose and increases the secretion of insulin, which increases the burden of islet *β* cells and causes cell damage and apoptosis. Insulin secretion is reduced after islet *β* cell damage, which eventually leads to T2DM.

The fasting glucagon level of patients with T2DM can be increased by 50% compared to those of nondiabetic patients [70]. There are two reasons for the increase in basal glucagon levels. One is that the high glucose state cannot inhibit the secretion of glucagon from pancreatic islet *α* cells so they continue to secrete glucagon [71]. The other is that *α* cells are not regulated by insulin, that is, insulin resistance exists [72].

S1P promotes the synthesis of GLP-1 by activating the PLC-PKC signaling pathway [65]. That is, S1P enhances the inhibitory effect of GLP-1 on glucagon secretion. Increased GLP-1 production plays an important role in maintaining the reactivity of islet *α* cells. GLP-1 can indirectly inhibit the secretion of glucagon by promoting the secretion of somatostatin by islet *δ* cells. GLP-1 can also directly inhibit glucagon secretion by islet *α* cells [73].

In addition, S1P alleviates insulin resistance in *α* cells in patients with T2DM. In the pancreatic islet *α* cells of T2DM patients, the insulin signaling pathways, such as Akt, are blocked, which leads to insulin resistance. When the S1P signaling pathway is activated, the Akt pathway can be activated to weaken insulin resistance [74].

### 3.7. S1P Improves the Imbalance of Glucose Processing in the Kidneys

The kidneys process glucose through glomerular filtration, proximal tubule reabsorption, and gluconeogenesis, which play an important role in glucose regulation [75]. Renal tubular glucose reabsorption is the most important mechanism for regulating blood glucose homeostasis. After culturing in a high-sugar environment, intracellular sodium-glucose co-transporter-2 mRNA and protein levels are significantly higher than in controls, and the reabsorption of glucose by the kidneys also increases significantly [76].

S1P protects and repairs glomerular and renal tubular cells. High glucose-induced proliferation of mesangial cells and accumulation of the extracellular matrix are the most important pathological changes in the early stage of diabetic kidney injury [77]. Studies have confirmed that S1P can promote glomerular hyperplasia [78]. In the diabetic nephropathy mouse S1PR_1_ agonist, the renal tubular epithelial cell barrier and relaxation function are improved; meanwhile, the damaged epithelial cells are impaired, all of which improve kidney microcirculation [79].

Otherwise, S1P reduces the inflammatory response of the kidney and prevents the occurrence of kidney damage under high glucose levels. The lack of S1PR_1_ in endothelial cells damages renal blood vessels and increases their permeability. Tubular necrosis, apoptosis, and inflammation are intertwined to exacerbate kidney injury [80, 81]. Therefore, S1P can protect the kidney from injury by reducing inflammation, weakening inflammation, and protecting renal proximal tubule cells [82].

### 3.8. S1P Regulates Neurotransmitter Function

The hypothalamus is an important center for regulating appetite and controlling glucose balance [83]. Obese individuals are characterized by insulin resistance and compensatory hyperinsulinemia. In obese and insulin-resistant patients, the degree of appetite suppression response decreases after glucose intake, and the time to reach the maximum suppression response is delayed. The patients' inhibitory effect on appetite in the hypothalamus is significantly weakened. Neurotransmitters, such as neuropeptides, reach the peripheral muscles, fat, and other tissues through body fluid transport to regulate glucose metabolism [84].

Depolarization of nerve cells induces the rapid generation of S1P. It is speculated that the increase in S1P concentration during depolarization may contribute to the release of norepinephrine and the increase of intracellular Ca^2+^ [85]. In addition, S1P is involved in the secretion of glutamate in hippocampal neurons through autocrine or paracrine means; nanomolar S1P causes glutamate secretion in hippocampal neurons, while picomolar S1P increases glutamate secretion. This indicates that S1P promotes neurotransmitter release [86]. Therefore, S1P enhances the function of neurotransmitters in the energy conversion of glucose in the liver, kidneys, muscle, and other organs.

## 4. Discussion

### 4.1. The Role of Sphingolipid Rheostat in Regulating T2DM

**Figure 3:**
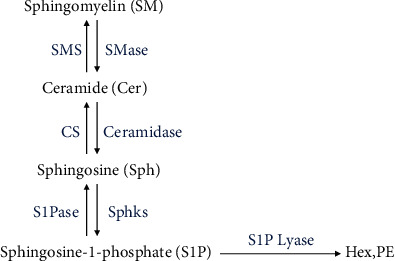
Metabolism of S1P in mammalian cells [87].

A possible reason for the contradicting function of S1P may be its relative levels instead of S1P directly determining the direction of cell and tissue metabolism [87]. The relevance of “sphingolipid rheostat” and its role in regulating T2DM have been carried out by numerous research groups using different cell types and experimental manipulations. The sphingolipid metabolites ceramide, sphingosine, and S1P constitute the S1P axis, which plays an important role in various organs, such as the pancreas and blood vessels. These metabolites are interconvertible (Figure 3) but usually have different functions. Active S1P stimulates growth and suppresses apoptosis [88], while ceramide and sphingosine usually inhibit proliferation and promote apoptosis [89–91]. On the onset of diabetes, not only should we pay attention to the impact of S1P but also the concentration of S1P in the circulation as well as the content of other molecules on the S1P axis. The combined effect of these factors is the effect of S1P.

This suggests that in the S1P research field, a specific S1PR agonist or antagonist should be selected to study the effects of S1PR_1-5_. If only S1P is used for physiological or pharmacological experiments, it will be activated at the same time, and the effects of different signal pathways will appear superimposed, affecting the authenticity and accuracy of the experimental results. When entering the stage of clinical drug research and development, attention should be paid to the development of selective S1PR ligands to try to avoid the adverse effects of S1PR_1_ and S1PR_2_.

### 4.2. Progress of Recent S1P Studies on Diabetes

S1P can directly promote *β* cell proliferation and inhibit apoptosis, indirectly reduce insulin resistance through adiponectin, and protect islets [25, 26, 92]. These findings confirm the beneficial effect of S1P on T2DM and provide a theoretical reference for promoting the application of S1P in clinical treatment. In addition, S1P can reduce mesangial cell inflammation through the SphK1 and S1PR_2_ pathways, reduce renal damage, and regulate glucose excretion, indicating that S1P also has a positive effect on the prevention and treatment of T2DM complications.

Studies regarding the S1PR_2_ signaling pathway in C2C12 skeletal muscle myoblasts suggest that the roles of S1PR may be diverse and even antagonistic to each other. The activation of S1PR_2_ can reduce the activity of protein-tyrosine phosphatase 1B, a major regulator of insulin receptor signal transduction, thereby promoting glucose uptake in skeletal muscle cells [93] and lowering blood glucose. The S1PR_1_ and S1PR_2_ signaling pathways may also have an effect on inhibiting glycometabolism and promoting insulin resistance. The SphK1/S1PR_1_/STAT3 axis may enhance and amplify insulin resistance [94]. Therefore, the role of S1P is not only beneficial for T2DM, especially in the activation of S1PR_1_ and S1PR_2_; it may destroy lipogenesis and promote the secretion of cytokines, thereby inhibiting the differentiation of fat cells and insulin signal transduction, inhibiting the proliferation of *β* cells and inducing their apoptosis. In addition, S1P has antiproliferative properties in rat liver cells by activating S1P2, suggesting that S1P is a negative regulator of liver regeneration. It cannot be excluded that the improvement of S1P-mediated insulin signal transduction is the result of changes in ceramide relative to the rheostat of S1P. Therefore, the role of SIP in hepatic insulin resistance is not yet fully understood [53, 95].

Furthermore, activation of specific S1PRs can inhibit adipose tissue production and promote lipolysis, which means higher free fatty acid concentration and more serious lipotoxicity. Jun et al. found that high concentrations of S1P can induce lipolysis through the cAMP PKA signaling pathway associated with S1PRs in cultured rat white adipocytes [96], which can also induce the downregulation of adipocyte-specific transcription factors, such as PPAR, C/EBP, and adiponectin, thereby ceasing adipogenesis and inhibiting lipid deposition [97]. The decomposition effect of S1P on adipose tissue mainly depends on the activation of S1PR1,2.

Jeong et al. found that in 3T3-L1 preadipocytes, adenovirus-mediated S1P2 overexpression can inhibit the JNK pathway and induce the downregulation of PPAR expression [98]. This indicates that the lipolysis of S1P may be mediated by the S1P2 and JNK signaling pathways.

FTY720, a S1PR agonist, inhibits adipogenesis and promotes lipolysis in DIO mice. The lipophilic effect of FTY720 phosphate involves increasing the phosphorylation of Ser563 and transcription of hormone-sensitive lipase, fatty triglyceride lipase, and perilipin [99]. In addition, FTY720 can promote insulin resistance in mature adipocytes and reduce glucose uptake [100].

### 4.3. Progress of Recent S1P Studies in Other Diseases

In addition to T2DM, recent studies regarding S1P have shown many interesting results in various areas.

FTY720/fingolimod, a functional S1PR1 antagonist, used clinically for nonpain conditions, is being utilized as nonnarcotic analgesics and may hopefully help reduce the abuse of opioids [101].

In March 2020, ozanimod capsules, agonists for S1PR1 and S1PR5, were approved for application in the treatment of relapsing forms of multiple sclerosis by the US FDA, involving clinically isolated syndrome, relapsing-remitting disease, and active secondary progressive disease in adults. In addition, CHMP recommended the approval of ozanimod in the EU for the therapy of relapsing-remitting multiple sclerosis with active disease defined by clinical or imaging features in adults. Furthermore, ozanimod has recently been assessed for its curative effect in ulcerative colitis and Crohn's disease, which has been undergoing multinational phase III trials [102].

Olesch et al. [103] found that the ablation of the immune cell-specific receptor S1PR4, which leads to increased CD8+ T cell abundance, delays tumor development and increases treatment success in murine models of breast carcinoma and colitis-associated colorectal cancer.

Regulating SphK, S1P, and S1PR pathways may have significant beneficial effects against acute and chronic life-threatening complications associated with SARS-CoV-2 infection [104].

### 4.4. Development of S1P-Related Drugs

FTY720 (fingolimod) is the first FDA-approved S1P-related drug. It is an immunomodulatory drug for treating multiple sclerosis that inhibits lymphocyte egress from lymphoid tissues by downregulating S1PR [105]. For FTY720, the main effect may be the internalization and continuous signal transduction of S1P1 [106].

However, S1P-related compounds have not yet been used to treat diabetes. Through the analysis of the role of S1P in the pathogenesis of diabetes, we found that S1P and its related molecules can increase the tissue's sensitivity to insulin, protect islet *β* cells, and regulate appetite, thus having the potential to be developed as drugs for T2DM. More importantly, if S1P is used as a drug, it may reduce the side effects caused by compound compatibility because S1P is an endogenous lipid molecule present in the human body.

## 5. Conclusion

The theory of “Ominous Octet” proposed by Professor Defronzo is a comprehensive conclusion on T2DM pathogenesis, and the role of S1P and its related metabolites in the development of T2DM has attracted increased attention. Therefore, we reviewed the role of S1P in each pathogenesis from the perspective of “Ominous Octet.” Overall, S1P plays an active role in preventing and delaying the development of T2DM.

The effects of S1P on T2DM can be summarized as follows: (1) S1P can promote *β* cell proliferation and antagonize its apoptosis; (2) S1P antagonizes insulin resistance in muscle tissue; (3) S1P enhances the Akt pathway and promotes hepatic glucose uptake and glycolysis, thereby reducing blood sugar; (4) it protects the liver and pancreas from lipotoxic damage; (5) it improves intestinal incretin effects; (6) it lowers basal glucagon levels; (7) it protects the barrier function of renal tubular epithelial cells and reverses kidney injury; (8) it promotes neurotransmitter release and transport to target organs, such as the liver, kidneys, and muscles to increase glucose utilization.

S1P is involved in multiple mechanisms of T2DM pathogenesis, and the broadness of its effects represents its great value in the diagnosis, prevention, and treatment of T2DM. However, the safety of S1P-related drugs for humans needs to be further investigated. The mechanisms of S1P and T2DM have not yet been fully elucidated, and further studies are needed both in laboratories and in clinics.

## Figures and Tables

**Table 1:** Signaling pathways mediated by S1PR_1-5_.

	Pathway	People	Time	Effect
S1PR_1_	Inhibit AC through Gi*α* [107]	Okamoto et al. [107]	1998	(1) Reduce cyclic adenosine phosphate (cAMP), promote cell proliferation, and increase survival/prevent cell apoptosis (2) Increase intracellular free calcium and promote insulin secretion at high glucose levels [108] (3) Promote erythrocyte migration, enhance endothelial barrier function and induce vasodilation, and reduce the occurrence of diabetic macrovascular complications [109]
	Activate PLC via Gi*βγ*, Ras-MAPK [107], PI3K/Akt [110]	Lee et al. [110]	1999	
S1PR_2_	Activate PI3K through Gi*βγ*, Ras	Kon et al. [111]	1997	(1) Promote cell proliferation, increase survival/prevent cell apoptosis, and increase intracellular free calcium (2) Inhibit migration, reduce endothelial barrier function, and induce vasoconstriction (3) Increase cyclic adenosine phosphate (cAMP) (4) Mediate the occurrence of diabetic retinopathy [112]
	Activate PLC via Gq			
	Activate Rho via G13			
	Activate AC [111], JNK, and p38 [23]	Gonda et al. [23]	1999	
S1PR_3_	Inhibit AC through Gi*α*	Okamoto et al. [113]	1999	(1) Reduce cAMP (2) Increase intracellular free calcium, promote cell proliferation, and promote insulin secretion at high glucose levels [108] (3) Reduce cytokine-induced islet *β* cell apoptosis [32, 114] (4) Promote endothelial cell migration and induce vasodilation (5) Mediate local inflammation of the kidney in diabetic nephropathy [17]
	Activate PLC, PI3K, and Ras via Gi*βγ*			
	Activate PLC via Gq			
	Activate Rho via G13 [113]			
	Activate AC [115]	Malek et al. [115]	2001	
S1PR_4_	Activation of Cdc 42 through Gi*βγ* [116]	Kohno et al. [116]	2003	(1) Mainly expressed in lymphoid tissue, lungs, the brain (especially oligodendrocytes), white blood cells, and the spleen [117]. The role is not clear.
	PLC [118]	Yamazaki et al. [118]	2000	
	ERK [119]	Van Brocklyn et al. [119]	2000	
	Activate Rho via G12/13 [38]	Graler et al. [120]	2003	
S1PR_5_	Inhibit AC through Gi*α* [121]	Im et al. [121]	2000	(1) Mainly expressed in lymphoid tissue, lungs, the brain (especially oligodendrocytes), white blood cells, and the spleen [117]. The role is not clear.
	Inhibit ERK, activate JNK, couple G12 [115]	Malek et al. [115]	2001	

## Data Availability

The data supporting this systematic review are from previously reported studies and datasets, which have been cited.
